# miR-34c-5p inhibited fibroblast proliferation, differentiation and epithelial-mesenchymal transition in benign airway stenosis via MDMX/p53 pathway

**DOI:** 10.1007/s10142-024-01317-y

**Published:** 2024-02-20

**Authors:** Jinmei Wei, Yan Chen, Tingmei Feng, Yuihui Wei, Caizhen Yang, Changwen Zhang, Wentao Li, Guangnan Liu

**Affiliations:** Department of Respiratory and Critical Care Medicine, The Second Affiliated Hospital of Guangxi Medical University, Nanning, China

**Keywords:** Benign airway stenosis, miR-34c-5p, Fibrosis, Epithelial-mesenchymal transition, MDMX, P53

## Abstract

Benign airway stenosis (BAS) means airway stenosis or obstruction that results from a variety of non-malignant factors, including tuberculosis, trauma, benign tumors, etc. In consideration of the currently limited research on microRNAs in BAS, this study aimed to explore the role and mechanism of miR-34c-5p in BAS. The expression of miR-34c-5p in BAS granulation tissues showed a significant down-regulation compared with the normal control group. Moreover, miR-34c-5p mimics suppressed the proliferation and differentiation of human bronchial fibroblasts (HBFs) and the epithelial-mesenchymal transition (EMT) of human bronchial epithelial cells (HBE). Conversely, miR-34c-5p inhibitors aggravated those effects. A dual-luciferase reporter assay confirmed that miR-34c-5p can target MDMX rather than Notch1. The over-expression of MDMX can reverse the inhibiting effect of miR-34c-5p on HBFs proliferation, differentiation and EMT. Furthermore, the expressions of tumor protein (p53) and PTEN were down-regulated following the over-expression of MDMX. In addition, the expressions of PI3K and AKT showed an up-regulation. In conclusion, miR-34c-5p was down-regulated in BAS and may inhibit fibroblast proliferation differentiation and EMT in BAS via the MDMX/p53 signaling axis. These findings expand the understanding of the role of miR-34c-5p and will help develop new treatment strategies for BAS.

**Supplementary Information:**

The online version contains supplementary material available at 10.1007/s10142-024-01317-y.

## Introduction

Benign airway stenosis (BAS) means airway stenosis or obstruction attributed to various non-malignant factors, including tuberculosis, trauma, benign tumors, etc. (Karagiannidis, et al. 2006). Previous studies have suggested that the genesis and progression of BAS are caused by airway mucosal inflammation, mucosal abnormal hyperplasia, epithelial-mesenchymal transition (EMT) and fibrous scar formation under the influence of various factors, such as infection, trauma and hypoxia, which leads to stenosis (Shi, et al. 2021; Wu, et al. 2021; Wei, et al. 2023). Owing to the action of cytokines like transforming growth factor-β1 (TGF-β1) following BAS, tracheobronchial fibroblasts aberrantly aggregate and proliferate, which results in the proliferation of granulation tissues and fibrosis (Wu, et al. 2021; Xu, et al. 2021). Given that the treatment of BAS is challenging due to a paucity of specific drugs and treatments, it is warranted to take effective measures during the early stage of tracheal injury to alleviate airway injury and the pathological repair process (Enyuan, et al. 2018).

As a highly conserved type of small non-coding ribonucleic acids (RNAs), microRNAs (miRNAs) play an essential role in cellular metabolism, cell proliferation and differentiation, EMT, tumorigenesis, fibrosis and other processes (Bartel 2004; Condrat et al. 2020). Considering that research on micro-RNAs in BAS is now limited, the role and mechanism of miR-34c-5p in BAS development was investigated in this study. MiRNA-34c-5p is a 23nt microRNA that inhibits fibrosis, extracellular matrix production, and cell proliferation and differentiation in various diseases by down-regulating target gene expression via binding to the 3’-untranslated region (3’-UTR) (Zhang, et al. 2022). For example, miR-34c-5p targets C-C motif chemokine ligand 22 (CCL22) to alleviate the development of chronic obstructive pulmonary disease (Gao et al. 2019). In addition, it inhibits EMT in cervical cancer by targeting Nocth1 (Wei et al. 2021), alleviates the occurrence and progression of femoral head osteonecrosis through mediating the murine double minute homolog 4/tumor protein (MDMX/p53) pathway (Yang, et al. 2022), and represses kidney fibrosis via calcium-calmodulin dependent protein kinase II (CaMKII) (Park et al. 2018).

For these reasons, it was speculated that miR-34c-5p may also play a crucial role in BAS. In this study, the expression level of miR-34c-5p in BAS tissues was explored, and its role and action mechanism were examined in cell models.

## Methods

### Clinical samples

The samples of BAS tissues were collected via bronchoscopy in the Department of Respiratory and Critical Medicine of the Second Affiliated Hospital of Guangxi Medical University (GXMU). The selection criteria of BAS patients were as follows: patients aged over 18 and diagnosed as BAS by medical history, bronchoscopy, chest computed tomography (CT) or pathology owing to various benign factors, such as endometrial tuberculosis, endotracheal intubation and tracheotomy. In addition, the exclusion criteria were as follows: patients aged under 18, with unknown or malignant etiology and insufficient data.

The samples of the normal control group were acquired from lung cancer patients who underwent surgical lobectomy in the Department of Cardiovascular Thoracic Surgery of the Second Affiliated Hospital of GXMU. The normal control patients were all aged over 18, and the removed bronchial tissue was pathologically confirmed to be free of tumor, inflammation or fibrosis, otherwise excluded.

Clinical samples above were placed into an RNA preservation solution at 4 ℃ overnight and stored at -80 ℃ for a long time. All operations gained the approval of the Ethics Committee of the Second Affiliated Hospital of GXMU [approval number: 2021-KY(0172)], and obtained the written consent of all the participants.

### Cell culture and treatment

Human bronchial epithelial (HBE) cells, human bronchial fibroblasts (HBFs) and human embryonic kidney cells 293T (HEK293T) cells were all offered by Wuhan Punosai Life Technology Co., Ltd. (Wuhan, China), and profiled by short tandem repeat (STR) analysis and negative mycoplasma. Cells were cultured in Dulbecco’s modified Eagle’s medium (DMEM, Gibco, Shanghai, China) high in glucose, and supplemented with 10% fetal bovine serum (FBS, Gibco), 100 µg/mL streptomycin (Solarbio, Beijing, China) and 100 IU/mL penicillin in a humidified incubator (5% carbon dioxide (CO_2_)) at the temperature of 37 ℃. Cells were observed and photographed with a microscope cell imaging system (Thermo Scientific).

### Cell transfection

MiR-34c-5p mimics, inhibitors, negative control (NC) and inhibitor NC miRNAs, and MDMX deoxyribonucleic acid (DNA) and blank control plasmids were all chemically synthesized by Suzhou GenePharma Co., Ltd. (Suzhou, China). Sequences are detailed in Table 1 and Supplementary Table 1. Lipofectamine 3000 (Invitrogen, Oregon (OR), the United States of America (USA)) was utilized for transfection as per the protocol of the manufacturer. The above-mentioned small interfering RNAs (siRNAs) were transfected into HBE cells or HBFs when cell confluency reached approximately 70-80%. The cell culture medium was replaced 24 h after transfection, and then TGF-β1 (the final concentration was adjusted to 10 ng/mL, Cloud-Clone, China) was used to stimulate the cells for 48 h.

**Table 1:** Sequences of siRNAs for transfection

Gene name	Sequence
MiR-34c-5p mimics-Forward	5’-AGGCAGUGUAGUUAGCUGAUUGC-3’
MiR-34c-5p mimics-Reverse	5’-AAUCAGCUAACUACACUGCCUUU-3’
MiR-34c-5p inhibitor	5’-GCAAUCAGCUAACUACACUGCCU-3’

### Cell counting Kit-8 assay

HBE cells and HBFs were treated with various concentrations of TGF-β1 or time interventions when cell confluency reached approximately 30-40% in 96-well plates. For transfection, equal amounts of cells and different siRNAs were respectively mixed evenly and planted in 96-well plates for 24 h, and continued to be cultivated for 48 h in the cell culture medium with TGF-β1. Cell Counting Kit-8 (CCK-8, NCM Biotech, China) was utilized for evaluating the cell viability of each sample by detecting optical density (OD) values by using a microplate reader.

### RNA extraction, RNA-sequencing and qRT-PCR

A Trizol kit (Takara, China) was employed to extract the total RNA from cells or tissues. A reverse transcription kit (Takara) was applied to synthesize complementary DNAs (cDNAs). Quantitative real-time polymerase chain reaction (qRT‑PCR) was conducted by the QuantStudioTM 5 Real-Time PCR System (Agilent Technologies) using the SYBR Green PCR Kit (Takara) as per the protocol of the manufacturer. U6 and glyceraldehyde-3-phosphate dehydrogenase (GAPDH) were taken as the internal references for miRNAs and mRNAs respectively. Additionally, 2^-△△CT^ was used as the relative expression level. Sangon Biotech (Shanghai, China) was employed to chemically synthesize primers whose sequences are presented in Table 2.

**Table 2:** Primer sequences for qRT-PCR

Gene name	Sequences
MiR-34c-5p-Forward	CGAGTGTAGTTAGCTGATTGCAAA
U6-Forward	GGAACGATACAGAGAAGATTAGC
U6-Reverse	TGGAACGCTTCACGAATTTGCG
MDMX-Forward	CTTCTCCGTGAAAGACCCAAGCC
MDMX-Reverse	TCCTGTGCGAGAGCGAGAGTC
Notch1-F	AAGAGTGCACCCATGGTACCAA
Notch1-R	TGACATGCATGATGCCTACATTTC
GAPDH-Forward	CTTTGGTATCGTGGAAGGACTC
GAPDH-Reverse	GTAGAGGCAGGGATGATGTTCT

### Western blot assay

A Radio Immunoprecipitation Assay (RIPA) buffer (Solarbio, Beijing, China) with a protease inhibitor mixture (Solarbio, Beijing, China) was used to lyse cells. A BCA Protein Assay Kit (Epizyme Biomedical Technology Co., Ltd, Shanghai, China) was utilized to quantify total protein. After being subjected to sodium dodecyl sulphate-polyacrylamide gel electrophoresis (SDS-PAGE), protein samples were electrotransferred to a polyvinylidene fluoride membrane (Millipore Company, USA). When 1-h blocking with 5% skim milk at ambient temperature was done, the membrane underwent overnight incubation with primary antibodies at 4 ℃. The rabbit primary antibodies used in this experiment were as follows: E-cadherin, collagen type I (COL-I), MDMX, B-cell lymphoma 2 (BCL-2), BCL2 associated X (Bax), PTEN, phosphoinositide 3-kinases (PI3K) and protein kinase B (AKT) (Proteintech, China), vimentin, alpha-smooth muscle actin (α-SMA), p53 and GAPDH (Servicebio, China). Next, the membrane underwent 1-h incubation with peroxidase-conjugated secondary antibodies [HRP Conjugated AffiniPure Goat Anti-rabbit IgG (H+L) (1: 5000, ProteinTech, China)] at ambient temperature after being washed. It was then placed in ECL™ Soak in the chemiluminescence detection reagent (NCM Biotech, China) for 1 min. Subsequently, the film was exposed to a gelatin film (GelView 6000M, Beijing, China) in a dark room for 30 s. The relative expression of proteins was determined by ImageJ (Rawak Software, Inc., Germany) and normalized to GAPDH.

### Dual-luciferase reporter assay

Targetscan 7.2 (https://www.targetscan.org/vert_72/)(Lewis, et al. 2005) and miRDB (https://mirdb.org/) (Chen and Wang 2020)were used to predict the target genes of miR‑34c‑5p. Afterwards, the targeting relationships between neurogenic locus notch homolog protein 1 (Notch1), MDMX and miR-34c-5p were verified by a dual-luciferase reporter assay. Dual-luciferase pSI‑Check2 vectors (Nocth1 3’ UTR and MDMX 3’ UTR wide and mutant types) for the dual-luciferase reporter assay were established by Suzhou GenePharma Co., Ltd. (Suzhou, China). Transfection reagents (Hanbio, China) were used to co-transfect HEK293T cells grown in a 96-well plate with miR-34c-5p mimic or NC and different dual-luciferase plasmid vectors for 6 hours. Luciferase activity which continued to culture the cells for 48 h was measured by performing a dual-reporter luciferase assay. The Luciferase Reaction Reagent was procured from Hanbio Science and Technology Co., Ltd. (Shanghai, China). Normalized luciferase activity was indicated by the ratio of Renilla to firefly luciferase activities (Rluc/fluc).

### Statistical analysis

Statistical Package for the Social Sciences (SPSS) v26.0 software (International Business Machines Corporation, IBM Corp.) was used to perform statistical analysis. A Student’s t-test or one-way analysis of variance was applied to compare two or more continuous variables, respectively. *χ*^*2*^ and Fisher’s exact tests were conducted to compare categorical variables. Kruskal-Wallis and Mann-Whitney tests were performed to compare non-parametric variables. Box plots, symbols, line plots and column charts were generated via the GraphPad Prism v8.0 (GraphPad Software, Inc.). It was considered that *P* < 0.05 showed statistical significance.

## Results

### Down-regulation of miR-34c-5p expression in BAS granulation tissues

**Fig. 1:**
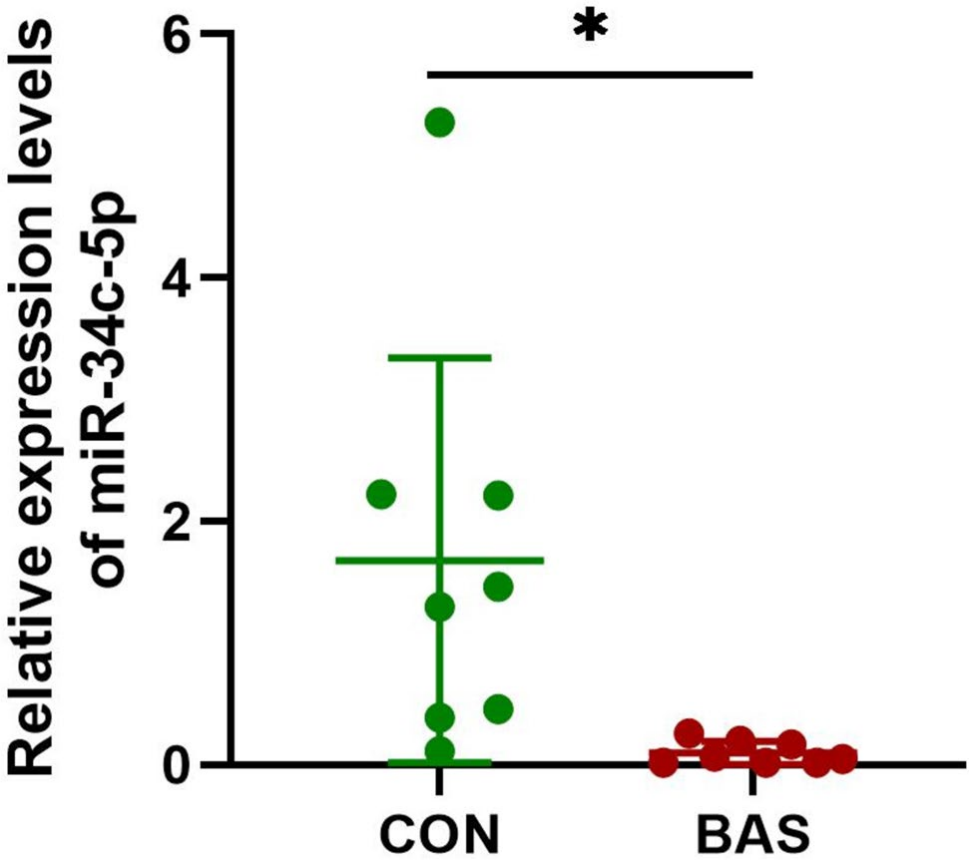
Relative expressions of miR-34c-5p in BAS granulation and normal tissues. CON: the normal control group, BAS: the benign airway stenosis, *P < 0.05

Tissues from 8 BAS patients (four males and four females in the age range of 21‑66, diagnosed with tracheal stenosis after intubation, tuberculous tracheobronchial stenosis and post-tracheotomy stenosis) were collected. Eight samples of the normal control group (four males and four females in the age range of 45‑74) were acquired. The baseline demographics and clinical features of these patients are listed in Supplementary Table 2. The expression of miR-34c-5p showed a significant (*P* < 0.05) down-regulation in BAS granulation tissues compared with the normal control group (Fig. 1).

### Inhibition of HBFs proliferation and differentiation by miR-34c-5p

**Fig. 2:**
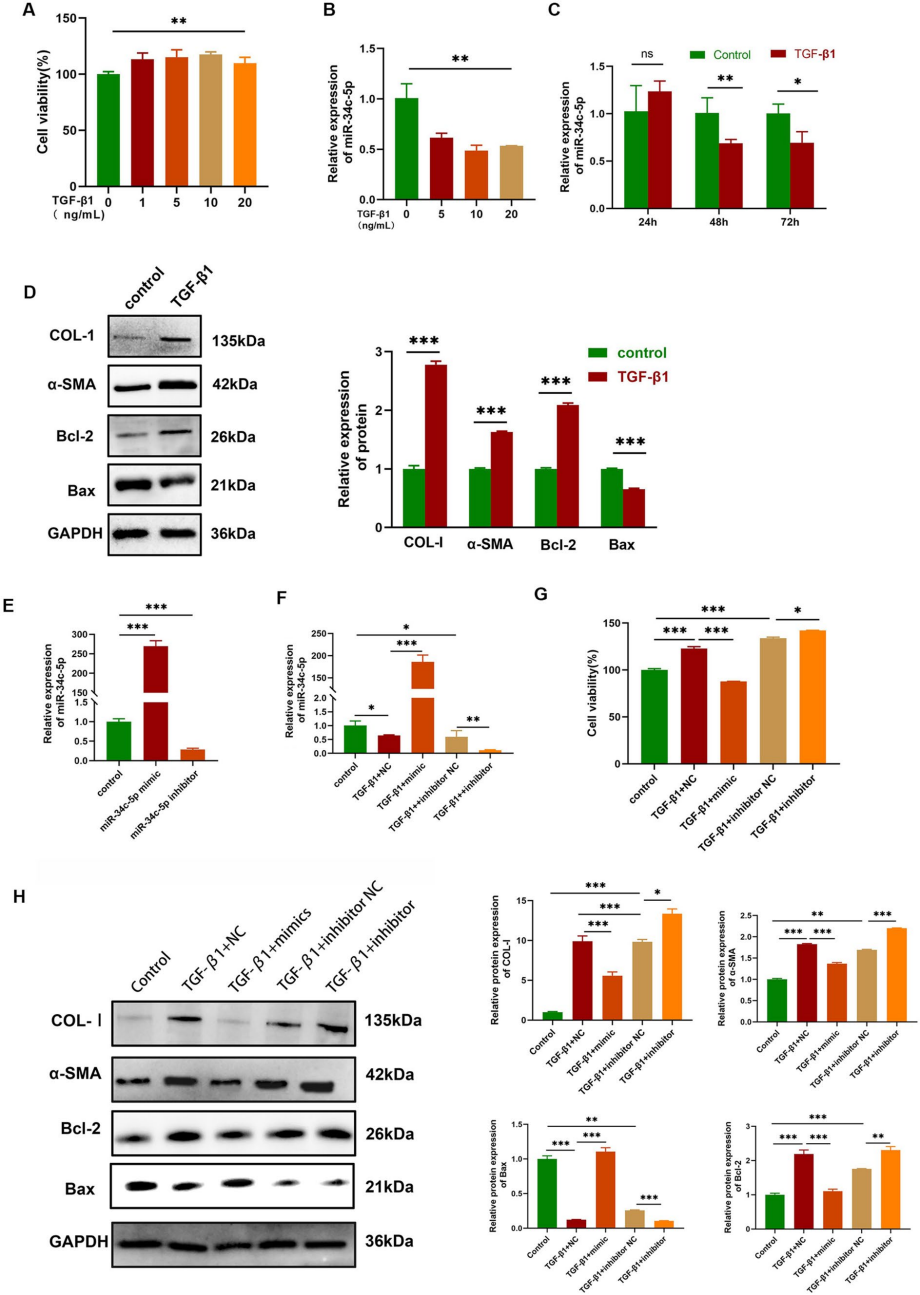
Inhibition of HBFs proliferation and differentiation by miR‑34c-5p. (**A**) Cell viability (%) of HBFs treated with various concentrations of TGF‑β1 by the CCK-8 assay. (**B**) Expression levels of miR-34c-5p in HBFs treated with different concentrations of TGF‑β1. (**C**) Expression levels of miR-34c-5p in HBFs treated with TGF‑β1 (10 ng/mL) at diversified time points. (**D**) Expression levels of COL-I, α‑SMA, Bcl-2 and Bax in TGF‑β1-stimulated HBFs by Western blot. (**E, F**) Expression levels of miR-34c-5p in HBFs that undergo transfection with different siRNAs. (**G**) Cell viability (%) of TGF‑β1-stimulated HBFs transfected with different siRNAs. (**H**) Expression levels of COL-I, α‑SMA, Bcl-2 and Bax in TGF‑β1-stimulated HBFs transfected with different siRNAs. TGF-β1 + mimics group: 24-h transfection with miR-34c-5p mimics, and then 48-h stimulation by TGF‑β1; TGF-β1 + NC group: 24-h transfection with NC miRNAs, and then 48-h stimulation by TGF‑β1; TGF-β1 + inhibitor group: 24-h transfection with miR-34c-5p inhibitors, and then 48-h stimulation by TGF‑β1; TGF-β1 + inhibitor NC group: 24-h transfection with inhibitor NC, and then 48-h stimulation by TGF‑β1. n = 3. Data were indicated by the mean ± standard error (SE), *P < 0.05, **P < 0.01 and ***P < 0.001

The CCK-8 assay revealed that cell viability was increased after treatment with 1, 3, 5, 10 and 20 ng/mL TGF‑β1 (*P* < 0.05) in comparison with the control group (Fig. 2-A). The relative expression level of miR-34c-5p in HBFs was stimulated with various concentrations of TGF-β1 or time points by qRT-PCR. The results indicated that the expression of miR-34c-5p showed a significant down-regulation compared with the control group and that the inhibiting effect was most pronounced when the concentration was 10ng/mL and time points were in 48 h (Figs. 2-B and 2-C). Western blot analysis confirmed that the expressions of COL-I, α-SMA and Bcl-2 increased, while the TGF-β1 group showed a decrease in the expression level of Bax compared with the control one (Fig. 2-D).

After HBFs were transfected with miR-34c-5p mimics and inhibitors and subsequently treated with TGF‑β1 (10 ng/mL) for 48 h, miR-34c-5p expression showed a significant up-regulation and down-regulation compared with the TGF‑β1 group, respectively (Figs. 2-E and F). The CCK-8 assay demonstrated that cell viability was decreased after transfection with the miR-34c-5p mimic, but increased after transfection with the miR-34c-5p inhibitor compared with the TGF-β1 group (Fig. 2-G). The miR‑34c‑5p mimic restored the expression of Bax and down-regulated the expressions of COL-I, α‑SMA and Bcl-2 compared with the TGF-β1 + NC group. Contrarily, the miR-34c-5p inhibitor decreased the expression of Bax and restored the expressions of COL-I, α‑SMA and Bcl-2 compared with the TGF-β1 + inhibitor NC group. All these results collectively signified that miR-34c-5p over-expression suppressed the proliferation and differentiation of HBFs, while miR-34c-5p down-expression exerted opposite effects (Fig. 2-H).

### Inhibition of HBE EMT by miR-34c-5p

**Fig. 3:**
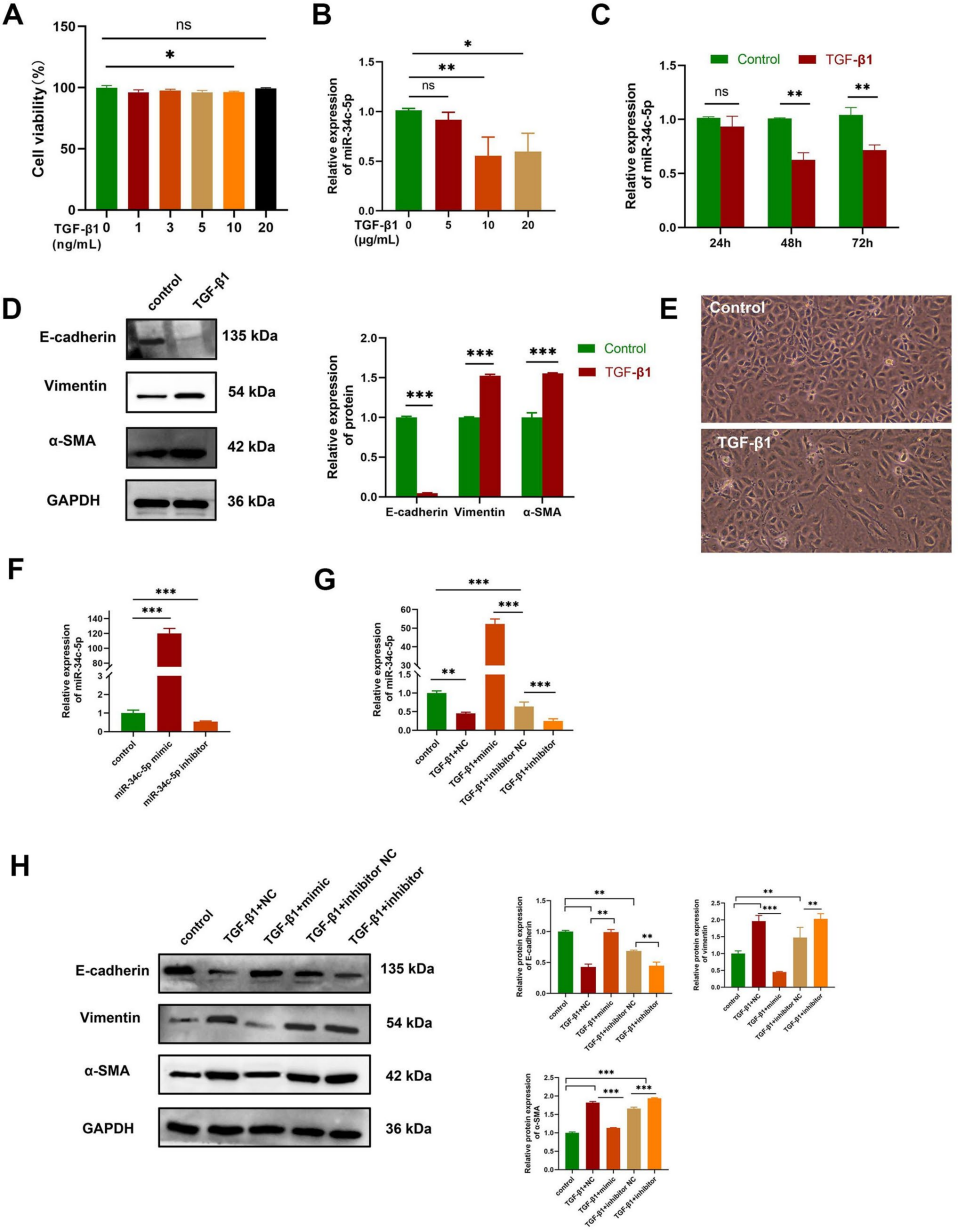
Inhibition of HBE EMT by miR-34c-5p. (**A**) Cell viability (%) of HBE cells treated with various concentrations of TGF‑β1 by the CCK-8 assay. (**B**) Expression levels of miR-34c-5p in HBE cells treated with different concentrations of TGF‑β1. (**C**) Expression levels of miR-34c-5p in HBE cells treated with TGF‑β1 (10 ng/mL) at diversified time points. (**D**) Expression levels of E-cadherin, vimentin and α‑SMA in TGF‑β1-stimulated HBE cells by Western blot. (**E**) Morphology of HBE cells under a microscope. Control: normal control, TGF-β1: treated by 10ng/mL TGF-β1 for 48 hours. (**F, G**) Expression levels of miR-34c-5p in HBE cells that undergo transfection with different siRNAs. (**H**) Expression levels of E-cadherin, vimentin and α‑SMA in TGF‑β1-stimulated HBE cells transfected with different siRNAs. TGF-β1 + mimics group: 24-h transfection with miR-34c-5p mimics, and then 48-h stimulation by TGF‑β1; TGF-β1 + NC group: 24-h transfection with NC miRNAs, and then 48-h stimulation by TGF‑β1; TGF-β1 + inhibitor group: 24-h transfection with miR-34c-5p inhibitors, and then 48-h stimulation by TGF‑β1; TGF-β1 + inhibitor NC group: 24-h transfection with inhibitor NC, and then 48-h stimulation by TGF‑β1. n = 3. Data was indicated by the mean ± standard error (SE), *P < 0.05, **P < 0.01 and ***P < 0.001, *ns:* no statistical significance

Cell viability showed a slight decrease after treatment by TGF‑β1 at concentrations of 1, 3, 5 and 10ng/ml by the CCK-8 assay compared with the control group (*P* < 0.05) (Fig. 3-A). In this study, qRT-PCR analysis was first performed to determine the relative expressions of miR-34c-5p in HBE cells that underwent 48-h stimulation with different concentrations (5, 10 and 20 ng/ml) of TGF‑β1. The expression levels of miR-34c-5p were significantly reduced by TGF‑β1 compared with the control group, and the inhibitory effect was optimal at a concentration of 10 ng/ml (Fig. 3-B). Next, the expressions of miR-34c-5p in HBE cells undergoing stimulation with 10 ng/ml of TGF‑β1 were determined at different time points (24, 48 and 72 h). It was found that the optimal inhibitory effect was achieved at 48 h (Fig. 3-C). Western blotting showed that TGF-β1 caused an increase in the expressions of vimentin and α-SMA and a significant decrease in the expression of E-cadherin (Fig. 3-D). These findings demonstrated that TGF‑β1 can induce EMT changes in HBE cells. Microscopical observation showed that HBE cells became elongated and tended to transform into mesenchymal cells after 48 hours of 10ng/mL TGF-β1 intervention compared with the control group (Fig. 3-E).

After HBE cells were transfected with miR-34c-5p mimics and inhibitors and subsequently treated with TGF‑β1 (10 ng/mL) for 48 h, miR-34c-5p expression showed a significant up-regulation and down-regulation compared with the TGF‑β1 group, respectively (Fig. 3-F and G). After HBE cells were transfected with miR-34c-5p mimics and NC and treated with TGF‑β1 (10 ng/ml) for 48 h, Western blot analysis revealed that TGF-β1 + NC and TGF-β1 + inhibitor NC groups showed an increase in the expressions of vimentin and α‑SMA and a reduction in E‑cadherin compared with the control one. MiR-34c-5p mimics suppressed the expressions of vimentin and α-SMA whereas increased the expression of E-cadherin compared with the TGF-β1 + NC group. On the contrary, miR-34c-5p inhibitors suppressed the expression of E-cadherin while increasing the expressions of vimentin and α-SMA compared with the TGF-β1 + inhibitor NC group. These findings indicated that EMT in HBE cells can be inhibited by miR‑34c‑5p mimics, but aggravated by miR‑34c‑5p inhibitors (Fig. 3-H).

### Target genes of miR-34c-5p

**Fig. 4:**
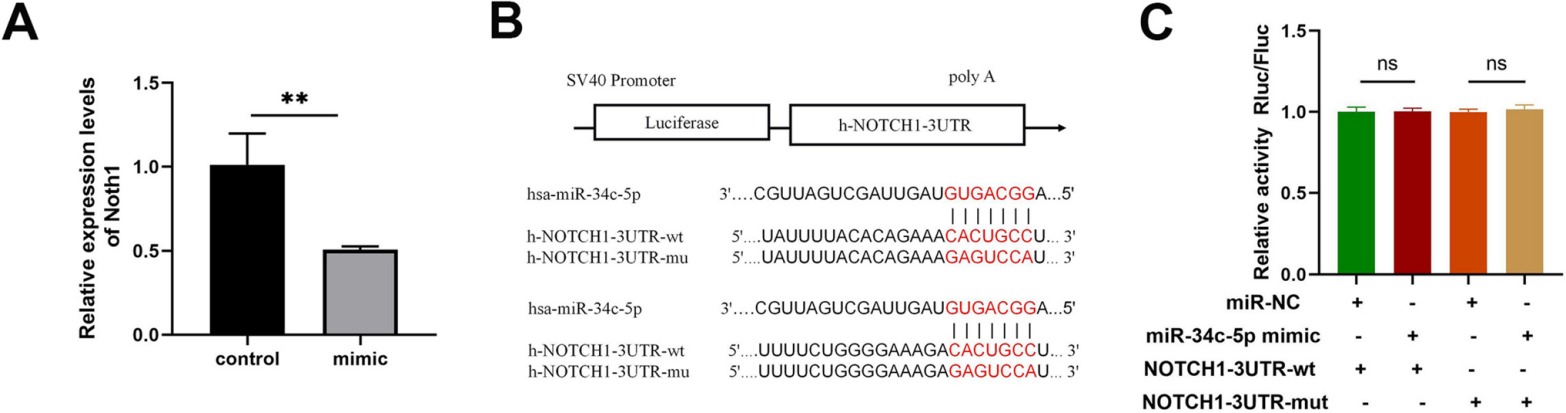
Nocth1 was not targeted by miR‑34c-5p. (**A**) Expression levels of Notch1 in NC and miR-34c-5p mimic groups determined by qRT-PCR and Western blot. Mimic group: 24-h transfection with miR-34c-5p mimics, and then 48-h culture. NC group: 24-h transfection with NC miRNAs, and then 48-h culture. (**B**) Sequence homology of the miR-34c-5p binding site in the Notch1 3’UTR. (**C**) Relative luciferase activity after the co-transfection of different siRNAs and dual-luciferase plasmids. n = 3. Data was indicated by the mean ± SE; *P < 0.05, **P < 0.01 and ***P < 0.001, *ns:* no statistical significance

It was predicted that Notch1 had binding sites with miR-34c-5p through Targetscan and miRDB (Supplementary Table 3). In addition, qRT-PCR confirmed that Nocth1 was down-regulated after being transfected with miR-34c-5p mimics (Fig. 4-A). Notch1 3’ UTR wild-type dual-luciferase (Nocth1-3' UTR-wt) and mutant plasmid vectors (Nocth1-3' UTR-mut) were constructed respectively (Fig. 4-B). However, the dual-luciferase reporter assay demonstrated that miR-34c-5p mimics could not reduce luciferase activity in wild-type or mutant plasmid groups (*P* > 0.05) compared with the NC group, which had no statistical significance (Fig. 4-C). These results showed that Notch1 could not be targeted by miR-34c-5p.

**Fig. 5:**
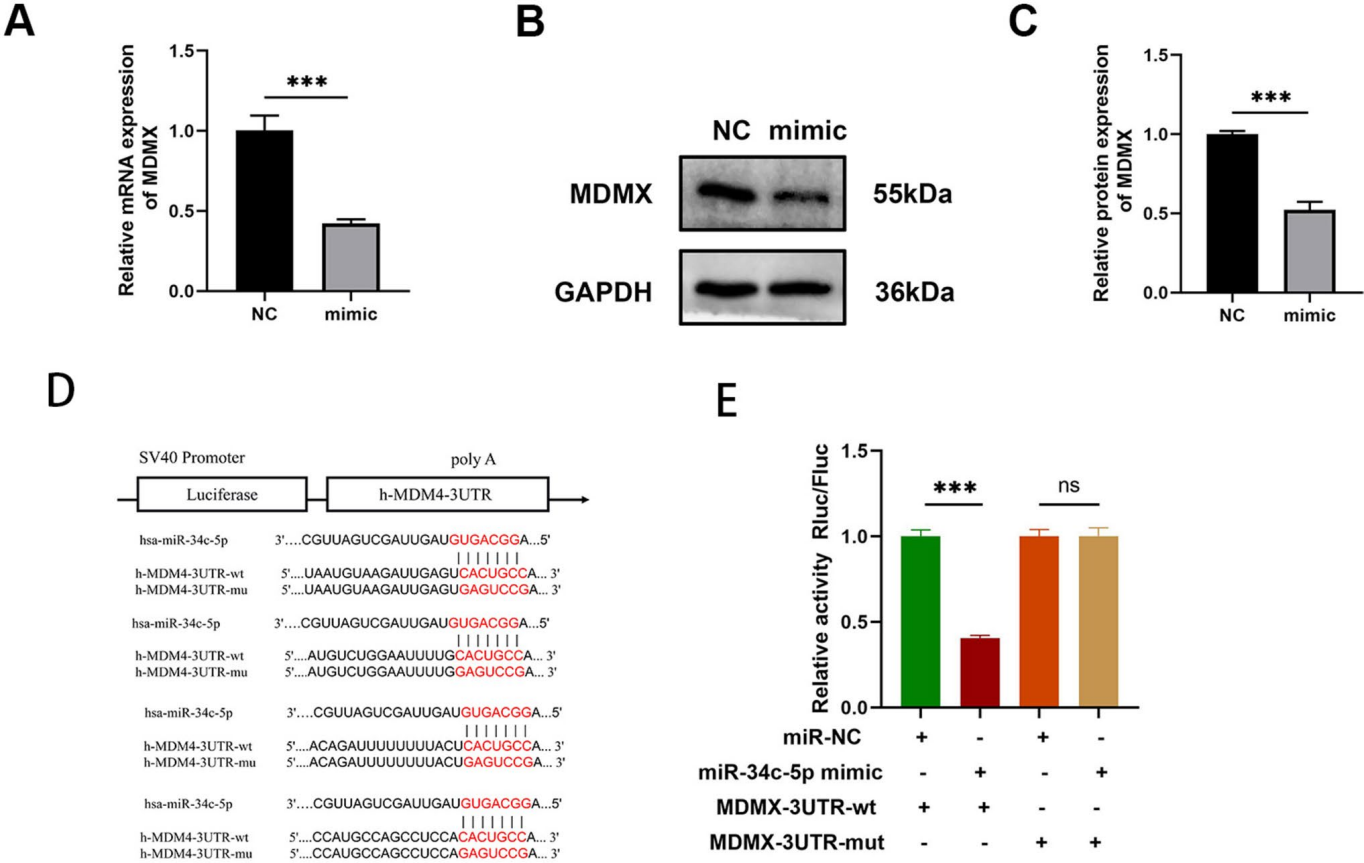
MDMX targeted by miR‑34c-5p. (**A**, **B, C**) Expression levels of MDMX in NC and miR-34c-5p mimic groups determined by qRT-PCR and Western blot. Mimic group: 24-h transfection with miR-34c-5p mimics, and then 48-h culture. NC group: 24-h transfection with NC miRNAs, and then 48-h culture. (**D**) Sequence homology of the miR-34c-5p binding site in the MDMX 3’UTR. (**E**) Relative luciferase activity after the co-transfection of different siRNAs and dual-luciferase plasmids. n = 3. Data was indicated by the mean ± SE; NS *P < 0.05, **P < 0.01 and ***P < 0.001, *ns:* no statistical significance

Then, it was predicted that MDMX contained binding sites with miR-34c-5p using Targetscan and miRDB (Supplementary Table 4). Furthermore, both qRT-PCR and Western blotting validated that MDMX showed a down-regulation after being transfected with miR-34c-5p mimics (Fig. 5-A and B). Thereafter, MDMX-3’ UTR-wt and MDMX-3’ UTR-mut were constructed (Fig. 5-C). The dual-luciferase reporter assay uncovered that miR-34c-5p mimics significantly lowered luciferase activity in the wild-type group (*P* < 0.05) rather than the mutant plasmid group (*P* > 0.05) compared with the NC group (Fig. 5-D). On balance, these results presented that miR-34c-5p could not target MDMX.

### Suppression of fibrosis by miR-34c-5p via the MDMX/p53 signaling pathway

**Fig. 6:**
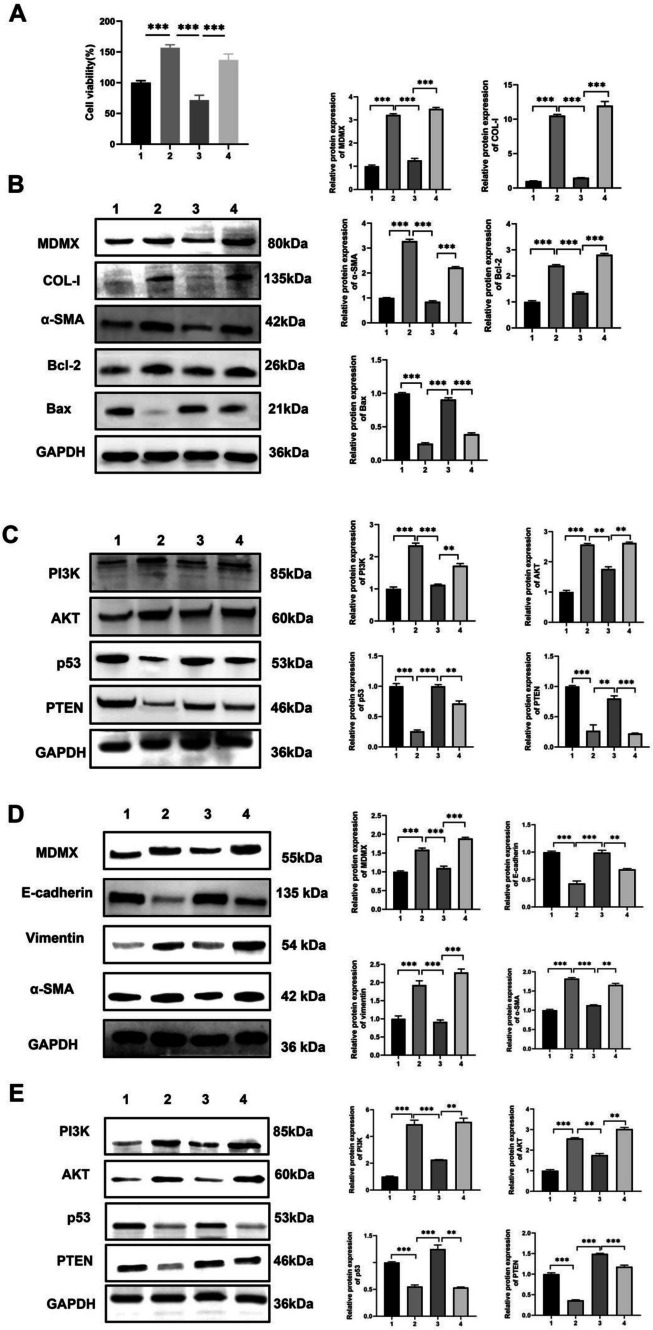
MDMX targeted by miR-34c-5p via the p53 pathway in HBFs and HBE cells. (**A**) Cell viability in different groups of HBFs. (**B**) Expression levels of MDMX, COL-I, α‑SMA, Bcl-2 and Bax in different groups of HBFs. (**C**) Expression levels of p53, PTEN, PI3K and AKT in different groups of HBFs. **(D)** Expression levels of MDMX, E-cadherin, vimentin and α‑SMA in different groups of HBE cells. **(E)** Expression levels of p53, PTEN, PI3K and AKT in different groups of HBE cells. Control and TGF-β1 groups (Groups 1 and 2): 24-h co-transfection with NC miRNAs and blank plasmids, and then 48-h stimulation by TGF‑β1; mimic group (Group 3): 24-h co-transfection with miR-34c-5p mimics and blank plasmids, and then 48-h stimulation by TGF‑β1; MDMX group (Group 4): 24-h co-transfection with miR-34c-5p mimics and MDMX-over-expressed plasmids, and then 48-h stimulation by TGF‑β1. n = 3. Data was indicated by the mean ± SE; *P < 0.05, **P < 0.01 and ***P < 0.001

MiR-34c-5p mimics were co-transfected with plasmids that overexpressed MDMX into HBFs and HBE cells to elucidate the mechanism by which miR-34c-5p inhibits fibrosis via targeting MDMX. The MDMX group showed an increase in cell viability and a down-regulation in the expressions of Bax, p53 and PTEN, but an up-regulation in the expressions of MDMX, COL-I, α‑SMA, Bcl-2, PI3K and AKT compared with the mimic one in HBFs model (Fig. 6-A, B, and C). In the HBE model, the MDMX group also down-regulated the expressions of E-cadherin, p53 and PTEN, while up-regulated the expressions of MDMX, vimentin, α‑SMA, PI3K and AKT compared with the mimic one (Fig. 6-D, and E). Overall, these results showed that the over-expression of MDMX could reverse the inhibiting effect of miR-34c-5p mimics on cell proliferation, differentiation and EMT.

## Discussion

Given that fibrosis plays an important part in the genesis and development of BAS, the inhibition of fibrosis can alleviate traumatic tracheal stenosis (Enyuan, et al. 2018; Xiao, et al. 2020; Wu, et al. 2021). Fibrosis is hallmarked by the aggregation of myofibroblasts, the proliferation of collagen, and the deposition of extracellular matrices. EMT plays a critical role in BAS. It is characterized by the decreased expression of epithelial adhesion proteins including E-cadherin and N-cadherin, which decreases the adhesion ability between cells, transformation into mesenchymal cells and further transformation into myofibroblasts that participate in the occurrence and development of fibrosis (Lamouille, et al. 2014; Duan, et al. 2016).

In the current study, the expression of miR-34c-5p showed a down-regulation in BAS granulation tissues. Earlier studies reported that down-regulating the expression of miR-34c-5p relieved lung, kidney and hepatic fibrosis (Li, et al. 2011; Disayabutr, et al. 2016; Park et al. 2018; Pang, et al. 2022), which indicated the possible role of miR-34c-5p in BAS-related fibrosis. In vitro, miR-34c-5p over-expression suppressed fibroblast proliferation, differentiation and EMT. Conversely, miR-34c-5p down-expression had the opposite effect, which insinuated that miR-34c-5p may alleviate fibrosis by suppressing fibroblast proliferation and differentiation. Both microscopic changes in the morphology of HBE cells and the decline in E-cadherin support the transformation of HBE cells into mesenchymal cells. This means that EMT may occur. However, it takes a long time for mesenchymal cells to transform into myofibroblasts, which needs to be confirmed by more research.

It was supposed that Notch1 may be the gene targeted by miR-34c-5p, which was confirmed by bioinformatics tools and PCR. Multiple studies have confirmed that miR-34c-5p inhibits EMT by targeting Notch1 in bladder cancer, cervical cancer and endometriosis (Xu, et al. 2019; Luo, et al. 2020). The Notch signaling pathway is a conserved communication system between adjacent cells because of its close relationship with cell proliferation and apoptosis. The inhibition of the Notch pathway can reduce renal fibrosis, retinal fibrosis, and lung fibrosis (Aoyagi-Ikeda, et al. 2011; Huang, et al. 2018; Fan, et al. 2020). However, the results of dual luciferase detection demonstrated that miR-34c-5p could not reduce the fluorescence values of Notch1 wild-type and mutant plasmids, and did not support the targeted regulation of Notch1 by miR-34c-5p. The dual luciferase results of this study were different from those of other studies, which may be related to plasmid construction, transfection processes and experimental methods.

In the meantime, the dual-luciferase reporter assay verified that MDMX can be targeted by miR-34c-5p. The over-expression of MDMX reversed the inhibiting effect of miR-34c-5p mimics on the proliferation and differentiation of HBFs. Besides, the expressions of p53 and PTEN were down-regulated following the over-expression of MDMX, while those of PI3K and AKT were up-regulated. Also referred to as HDMX or MDM4, MDMX is a regulator of p53 and co-regulates p53 expression with MDM2 (Wade, et al. 2013). The p53 protein is highly involved in cell proliferation, apoptosis, cycle and senescence, EMT, DNA damage repair, autophagy, etc. (Lu et al. 2020). Importantly, it promotes cell apoptosis by directly down-regulating the expression of Bcl-2 and concurrently up-regulating those of Bax and caspase-3 (Kastenhuber and Lowe 2017). A mutual regulatory relationship exists between p53 and miRNAs. Indeed, p53 expression is directly governed by some miRNAs, while regulated by others via targeting MDM2 or MDM4. p53 also regulates a group of miRNAs like the miR-34 family (Rokavec, et al. 2014; Liu, et al. 2017). Recent research has established that miR-34c-5p alleviates the genesis and progression of femoral head osteonecrosis by mediating the MDMX/p53 pathway (Yang, et al. 2022) and inhibiting pulmonary fibrosis via p53 and PTEN/PI3K/Akt signaling pathways by targeting Fos-related antigen 1 (Fra-1) (Pang, et al. 2022).

The PTEN protein is a downstream target of p53 that participates in cell proliferation, apoptosis and EMT (Lee, et al. 2018; Tian, et al. 2019). In addition, it exerts biological effects by inhibiting PI3K phosphorylation and thereby the PI3K/AKT pathway, which is central to fibrosis, inflammation and immune regulation (Sun, et al. 2021; Wang, et al. 2022). Also, this study corroborated the function of PTEN and the PI3K/AKT signaling pathway in the proliferation and differentiation of fibroblasts.

**Fig. 7:**
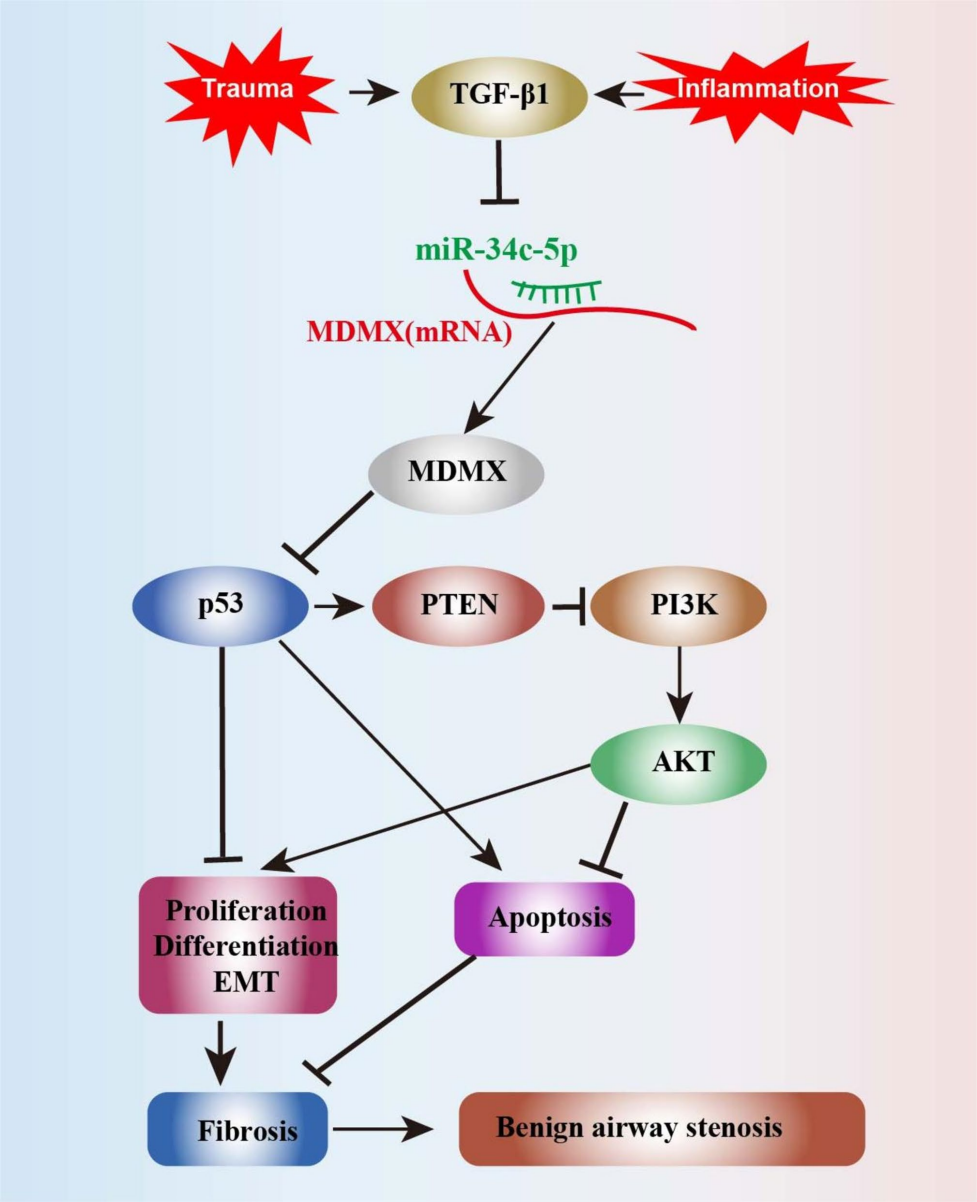
Action mechanism of miR-34c-5p in BAS

Herein, the expressions of MDMX, PI3K and AKT were down-regulated, while those of p53 and PTEN were up-regulated after miR-34c-5p mimics were transfected into HBFs. This indicates that miR-34c-5p mimics can inhibit the PI3K/AKT signaling pathway by up-regulating the expressions of p53 and PTEN. After miR-34c-5p mimics were co-transfected with plasmids overexpressing MDMX, the expressions of MDMX, PI3K and AKT were up-regulated, while those of p53 and PTEN were down-regulated. That suggests MDMX-over-expressed plasmids could reverse the promotion effect of miR-34c-5p mimics on the expression of p53/PTEN. It was postulated that miR-34c-5p may potentially suppress the proliferation and differentiation of HBFs by inhibiting MDMX expression in p53 and downstream PTEN/PI3K/AKT signaling pathways (Fig. 7).

To sum up, this research demonstrated that miR-34c-5p expression showed a down-regulation in BAS, and miR-34c-5p over-expression inhibited fibroblast proliferation and differentiation in vitro. More importantly, miR-34c-5p targets MDMX for regulating p53 and PTEN\PI3K\AKT signaling pathways. However, further in vivo studies are necessitated to ascertain the role played by miR-34c-5p in attenuating BAS by inhibiting fibrosis.

## Conclusion

MiR-34c-5p expression showed a down-regulation in granulation tissues with BAS, which signaled the close association between miR-34c-5p and BAS. Herein, miR-34c-5p was confirmed to have the ability to inhibit the in vitro proliferation and differentiation of bronchial fibroblasts. The dual-luciferase assay demonstrated that miR-34c-5p can target MDMX. Besides, the over-expression of MDMX could reverse the inhibiting effect of miR-34c-5p on the proliferation and differentiation of fibroblasts. In conclusion, miR-34c-5p relieves fibrosis through the MDMX/p53 signaling pathway in BAS. All these findings advance the understanding of the role of miR-34c-5p in designing new therapeutic strategies for treating BAS.

### Supplementary Information

Below is the link to the electronic supplementary material.

Supplementary file1 (XLSX 9 KB)

Supplementary file2 (XLSX 10 KB)

Supplementary file3 (XLSX 272 KB)

Supplementary file4 (XLSX 850 KB)

## Data Availability

The data used and analyzed during the current study is available from the corresponding author upon reasonable request.
